# CRISPR-Cas9 as a Powerful Tool for Efficient Creation of Oncolytic Viruses

**DOI:** 10.3390/v8030072

**Published:** 2016-03-07

**Authors:** Ming Yuan, Eika Webb, Nicholas Robert Lemoine, Yaohe Wang

**Affiliations:** 1 Centre for Molecular Oncology, Barts Cancer Institute, Queen Mary University of London, London EC1M 6BQ, UK; m.yuan@qmul.ac.uk (M.Y.); e.s.webb@smd12.qmul.ac.uk (E.W.); bci-director@qmul.ac.uk (N.R.L.); 2 National Centre for International Research in Cell and Gene Therapy, Sino-British Research Centre for Molecular Oncology, Zhengzhou University, Zhengzhou 450052, China

**Keywords:** Oncolytic virus, CRISPR-Cas9, homologous recombination, Vaccinia virus, adenovirus, Herpes simplex virus

## Abstract

The development of oncolytic viruses has led to an emerging new class of cancer therapeutics. Although the safety profile has been encouraging, the transition of oncolytic viruses to the clinical setting has been a slow process due to modifications. Therefore, a new generation of more potent oncolytic viruses needs to be exploited, following our better understanding of the complex interactions between the tumor, its microenvironment, the virus, and the host immune response. The conventional method for creation of tumor-targeted oncolytic viruses is based on homologous recombination. However, the creation of new mutant oncolytic viruses with large genomes remains a challenge due to the multi-step process and low efficiency of homologous recombination. The CRISPR-associated endonuclease Cas9 has hugely advanced the potential to edit the genomes of various organisms due to the ability of Cas9 to target a specific genomic site by a single guide RNA. In this review, we discuss the CRISPR-Cas9 system as an efficient viral editing method for the creation of new oncolytic viruses, as well as its potential future applications in the development of oncolytic viruses. Further, this review discusses the potential of off-target effects as well as CRISPR-Cas9 as a tool for basic research into viral biology.

## 1. Introduction

Oncolytic viruses (OVs) are anti-tumor viruses that selectively infect and kill cancer cells without damaging normal tissues [1]. They can also elicit anti-tumor immune responses to specifically kill uninfected cancer cells [2]. Over the past two decades, there has been mounting evidence that OVs are effective in treating cancer in both preclinical models and clinical trials [2,3,4,5]. The most tested OVs in preclinical and clinical trials are the Herpes simplex virus (HSV), Vaccinia virus (VV), and adenovirus (AdV). In one study, Talimogene laherparepvec (also called T-VEC), an oncolytic HSV coding for granulocyte/macrophage-colony stimulating factor (GM-CSF), was administered by direct intratumoral injection to patients with metastatic malignant melanoma and this led to complete regressions of injected and uninjected lesions in eight of 50 patients [2]. In addition to single therapy, HSV-GMCSF (T-VEC) has also been used in combination with radiotherapy and cisplatin in clinical trials to treat stage III/IV head and neck cancer [6]. An oncolytic vaccinia virus (JX-594) armed with GM-CSF, showed promising results in preclinical and clinical trials treating liver cancers [5,7]. A range of Adenoviruses has been explored extensively as viral vectors for gene therapy and also as oncolytic viruses. Thus, so far, of the many different OVs being investigated for treating cancers, the genetically-modified adenovirus H101 is the first oncolytic virus to be accepted, receiving Chinese FDA approval in 2005 for the treatment of head and neck cancer [8,9].

Modifications of viral genomes have been made to render OVs more selective to cancer cells. The deletion of thymidine kinase (TK) in OncoVEX and JX594 increases their selectivity to cancer cells, while H101 lacking the E3B-55kDa gene replicates selectively in cancer cells. Further, minimizing the host immune response represents an important target for cancer therapeutics [10], which can be achieved by arming the oncolytic virus with therapeutic cytokines enhancing the anti-tumor immune response. Oncolytic VV JX-594 and oncolytic HSV OncoVEX, both armed with GM-CSF, show clinical benefits can be achieved through localized oncolytic activity, as well as a systemic anti-tumor immune response [2,11,12].

Thus, viruses have become important potential vectors for cancer therapy. The conventional method for generating tumor-targeted OVs with large genomes (such as vaccinia virus, HSV, and adenovirus) is based on homologous recombination using a shuttle vector, either in bacteria or in mammalian cells. Currently, there are three major established methods which are used to edit the adenovirus genome: a bacteria-based homologous recombination system [13,14], a bacterial artificial chromosome (BAC) system [15], and a hybrid yeast–bacteria cloning system [16]. These systems require laborious multi-step methods with low efficiency and can be tedious. The popular method for modification of VV is based on homologous recombination using a repair donor DNA shuttle vector. Unfortunately, this process has a homologous recombination efficiency of less than 1% and often the selection marker is randomly inserted elsewhere into the VV genome and, subsequently, drops out upon virus expansion [17,18]. Recombinant HSV-1 vectors have been constructed using homologous recombination techniques, which require time-consuming selection processes and structural confirmation. The BAC system was also used to develop mutant oncolytic HSV-1, requiring manipulation of plasmids in bacteria and virus packaging in host cells [19]. Thus, in the interests of time and convenience, it would be beneficial to develop a more efficient and straightforward method for editing large viral genomes to construct mutant or recombinant DNA viruses [20,21].

The CRISPR (clustered regularly interspaced short palindromic repeat)-Cas system is a naturally-evolved adaptive immune system targeted against invading phages and other genetic elements in bacteria and archaea [22,23,24]. There are five types of CRISPR systems (I-V) in a range of microbial species [25], of which the type II CRISPR-Cas system has generated the most interest. Under the guidance of RNA-guided Cas9 endonuclease derived from *Streptococcus pyogenes*, a single guide RNA (sgRNA) and the trans-activating crRNA (tracrRNA), the type II CRISPR-Cas9 system has dramatically altered the way genomes are being edited in eukaryotic cells [26,27]. The CRISPR-Cas9 system has been engineered to cleave any sequence preceding a 5′-NGG-3′ protospacer adjacent motif (PAM) sequence in mammalian cells [26,27]. PAM is a DNA sequence immediately following the DNA sequence targeted by the Cas9 nuclease. Since its initial application in human cells in 2013 [26,27,28,29], CRISPR-Cas9 has been adapted for genomic editing in mammalian cells and this has been utilized in a multitude of ways. For example, CRISPR-Cas9 has been used *in vivo* to generate genetically engineered mice for the study of human diseases [30] and for the induction of genomic alterations in plants [31], zebrafish [32], *Drosophilia* [33], *Caenorhabditis elegans* [34], and yeast [35]. Recently, it has been successfully employed in manipulating the genomes of various viruses, including HSV, VV, and AdV [18,36,37]. Here we focus on the application of CRISPR-Cas9 system in the engineering of mutant OVs.

## 2. Viral Genome Editing Using the CRISPR-Cas9 System

Enhanced Green Fluorescent Protein (EGFP) in a recombinant adenoviral vector (AdV-EGFP) was used as the target gene to be edited by Cas9, directed by three alternative guide RNAs [36]. Cas9 guided by all three gRNAs could induce mutations in the EGFP gene. The efficiency of induced mutations was as high as 47.4% with the guide RNA construct gRNA-175. The high efficiencies of mutations in the target region of gRNAs within the genome of adenovirus were further confirmed by DNA sequencing and these mutations could be passed onto progeny viruses upon infection of cells with the p1 virus. The non-homologous end joining (NHEJ) mechanism can be used to repair the adenovirus DNA genome following targeted site cleavage by Cas9 [36].

The mutation efficiency induced by the CRISPR-Cas9 system was examined relative to the expression of Cas9 and gRNA. The mutation efficiency reached its peak between 24 and 36 h post-transfection of Cas9 and gRNA. The amount of virus used to infect cells also affects the mutation efficiency, showing the highest levels when the MOI used was between 1 and 10.

The CRISPR-Cas9 system was more efficient than the transcription activator-like effector nuclease (TALEN) technology when targeting the same region of the AdV genome [36]. Furthermore, the CRISPR-Cas9 system has been used successfully to delete a fragment of DNA by simultaneously targeting two sites using two gRNAs, where the DNA deletion was confirmed by PCR and further sequencing [36].

To test the ability of the CRISPR-Cas9 system to edit the genome of HSV1, thymidine kinase (TK) was targeted by the guide RNA vector gRNA-206 [36]. The HSV1 virus with inactivation of the deleted-TK gene is resistant to antiviral drug acyclovir (ACV) [38], therefore ACV has been employed to select the mutant HSV1 virus with the deficient function of TK gene [39]. ACV-resistant viruses were isolated from the cells transfected with Cas9 and gRNA-206, to select for the inactivation of the TK gene by gRNA-guided Cas9. The ACV-resistant viral progeny made up 50.1% of the total virus, indicating a high efficiency of mutation induced by CRISPR-Cas9 in the TK gene, and DNA sequencing confirmed that all ACV-resistant progeny viruses contained the mutation in the TK gene. Suenaga *et al.* reported that 60% of the clones contained the expected mutations in the gE gene of HSV1 and 50% of the clones contained the expected mutations in the TK gene of HSV1, determined by DNA sequencing [40].

Unlike AdV and HSV1, which replicate in the nucleus, vaccinia virus replicates in the cytoplasm, and indels formed in the target region of N1L and A46R of VV were lower than 10%. This could be due to the lower repair efficiency of the NHEJ mechanism in the cytoplasm [18].

## 3. CRISPR-Cas9 System Induces High Efficiency of Homologous Recombination

**Figure 1:**
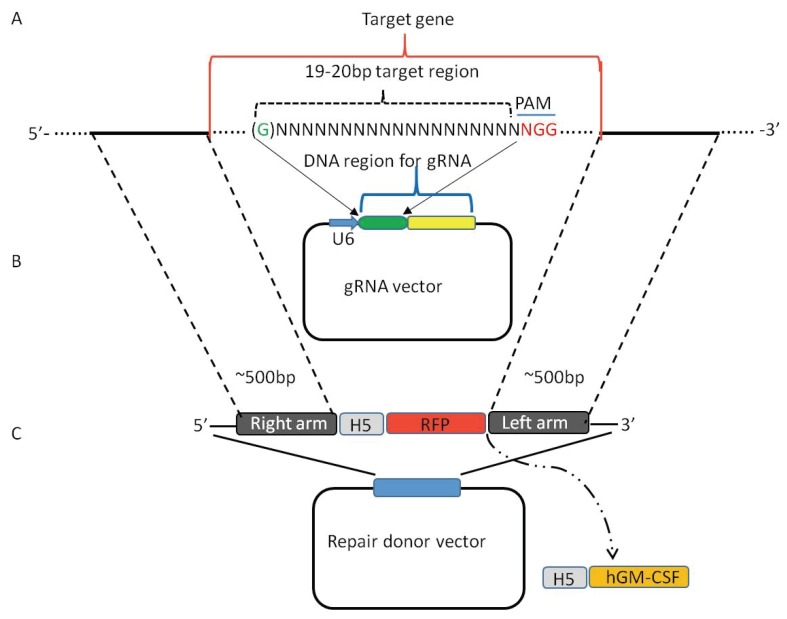
Construction of gRNA vector and repair donor vector for creation of a mutant vaccinia virus. (**A**) A 19mers or 20mers gRNA target sequence with/without a G at 5′ end is designed within the target gene; (**B**) DNA sequence of a gRNA target region is cloned into a gRNA vector using U6 at the promoter; and (**C**) a repair donor vector is constructed. The length of right arm and left arm is about 500 bp, both arms can just flank the target gene, or slightly overlap with target gene up to 50 bp. The purification marker RFP driven by the vaccinia virus promoter H5 is cloned between the right arm and left arm in the donor vector, a therapeutic gene driven by H5 promoter, such as human granulocyte-macrophage colony-stimulating factor (hGM-CSF), can be cloned into the site between RFP and the left arm. The RFP or a therapeutic gene does not need a poly A signal to stabilize the mRNA as the mRNA is transcribed in the cytoplasm.

**Figure 2:**
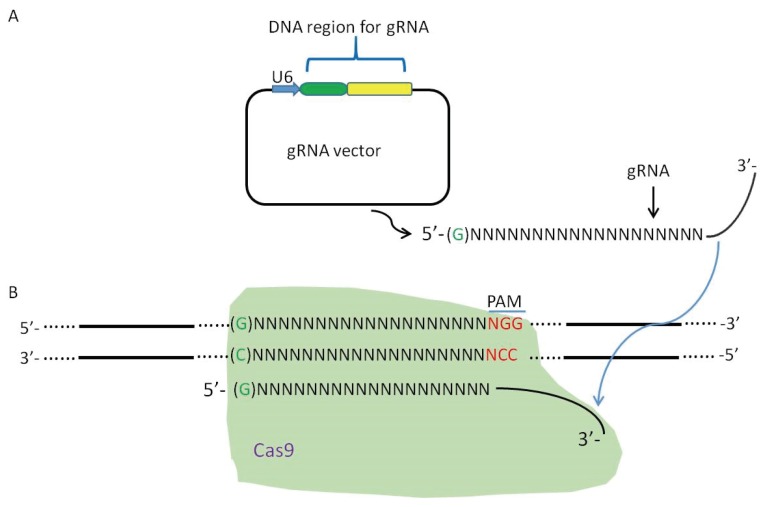

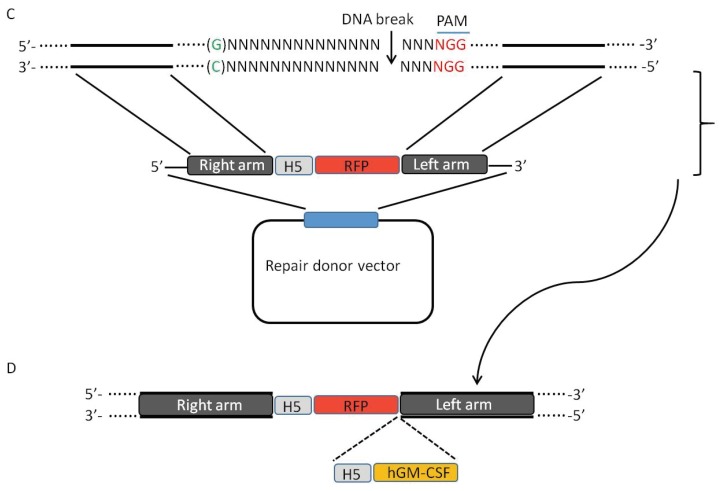
gRNA guided Cas9 induces homologous recombination by creating a DNA double-stranded break in the target region of vaccinia virus. (**A**) The gRNA is transcribed; (**B**) gRNA guides Cas9 to the target site; (**C**) Cas9 creates a DNA double-stranded break, which is repaired by the repair donor vector via the mechanism of homologous recombination; and (**D**) the mutant vaccinia virus is generated with the deletion of the target gene and insertion of the purification marker RFP containing its promoter H5. A therapeutic gene hGM-CSF and its promoter H5 can also be incorporated into the target gene.

Generation of mutant vaccinia viruses mainly relies on homologous recombination to delete a particular target gene, or to arm the virus with a gene in the target region [14,41,42,43,44,45]. DNA double-stranded breaks can effectively induce homologous recombination in mammalian cells [46] and this mechanism can be harnessed to improve efficiency in the generation of VV mutants (Figure 1 and Figure 2). The CRISPR-Cas9 system has been successfully employed in improving homologous recombination in eukaryotic organisms [26,47]. The CRISPR-Cas9 system has been used to generate mutant viruses with greater efficiency including AdV, HSV1 and VV mutants. This system can induce homologous recombination with efficiencies up to 2%–3% in introducing the DsRed gene into the AdV genome, where whole-genome sequencing of mutant AdV confirmed the incorporation of the DsRed gene into the target region and also that no off-target mutations were induced [18,36,37]. To test the ability of the D10A nickase mutant of Cas9 in inducing mutations in viral genome, a single gRNA or a pair of gRNAs were used to induce homologous recombination. The results suggest that a nickase guided by a single gRNA combined with a repair donor DNA can generate more precisely targeted viral mutants [36].

A mutant HSV1 expressing the EGFP reporter gene was constructed by combining gRNA-guided Cas9 and a homologous repair donor DNA. This increased the efficiency of homologous recombination from less than 0.00000145% of total plaques using control cells (without transfection of gRNA-guided Cas9) to 8.41% of total plaques using cells transfected with gRNA, Cas9, and a repair donor DNA. A HSV-1 gE-revertant virus was successfully generated using the CRISPR-Cas9 system, and the frequency of His-tag knock-in viruses in the gE region was around 10%, confirmed by PCR and subsequent sequencing [40].

As VV replicates in the cytoplasm, a plasmid encoding Cas9 without a nuclear localization signal for editing the vaccinia virus genome was developed by our group, the efficiency of homologous recombination in the reporter gene-positive plaques was as high as 85% in the A46R region, 62.5% in the N1L region [18] and 94% in the TK region [37]. Compared to the efficiency of conventional homologous recombination (less than 1%), this is a greater than 50-fold increase. Impressively, two genes of VV were modified simultaneously by combining a gRNA targeting the N1L region and a gRNA targeting the A46R region, and the efficiency of double homologous recombination was 60% [18]. A marker-free mutant VV with deletion of the TK and N1L genes was also created efficiently using the CRISPR-Cas9 system combined with Cre-Loxp and Flp-FRET systems [37].

## 4. Off-Target Effects by CRISPR-Cas9

As CRISPR-Cas9 is increasingly being employed for various uses, there is a growing concern over its target specificity and off-target effects. Five mismatches between the gRNA and the complementary target sequence, especially at the 5′ end of gRNA, can be tolerated by Cas9 while the stringency of the 3′ end of gRNA is crucial for the integrity of Cas9 [47]. CRISPR-Cas9 has shown substantial off-target effects at sites with similarity to the gRNAs in cultured cells [28,48,49]. Various strategies have been developed to reduce off-target effects induced by Cas9. One approach is to use the truncated sgRNAs bearing shortened regions of target site complementarity [50,51]; Another strategy to minimize the potential for off-target errors, is to use the Cas9 nickase mutant D10A as it requires a pair of gRNAs to create double-strand breaks at the target region [52,53]. Furthermore, the dimeric fusions of catalytically-inactive Cas9 to a non-specific FokI nuclease has been used as well to reduce the off-target effects by Cas9 [54,55,56]. However, these strategies may partially effective, have as-yet unproven efficacies on a genome-wide scale, and/or possess the potential to create more new off-target sites. Recently, Joung’s lab developed a robust and easily used strategy that eliminates off-target mutations on a genome-wide scale using a high-fidelity CRISPR-Cas9 nucleases (called SpCas9-HF1) [57]. SPCas9-HF1 is a high-fidelity variant harboring alterations designed to reduce non-specific DNA contacts. SpCas9-HF1 keeps on-target activities comparable to wild-type SpCas9 tested in human cells. Notably, with sgRNAs targeted to standard non-repetitive sequences, the SpCas9-HF1 does not induce off-target events detectable by genome-wide break capture and targeted sequencing methods. Strikingly, even for atypical, repetitive target sites, the vast majority of off-target mutations induced by wild-type SpCas9 were not detected with SpCas9-HF1. SpCas9-HF1 provides an alternative to wild-type SpCas9 for research and therapeutic applications.

Off-target effects of Cas9 were examined in a series of experiments in which the adenovirus AdV-EGFP was edited using the CRISPR-Cas9 system. Upon analysis of the gRNA-175 sequence against the adenovirus genome, there were no significant homologous sequences found. Whole-genome sequencing of the AdV-EGFP mutant generated using Cas9 directed by gRNA-175 revealed only one guanine deletion in the gRNA-175 target site, and this suggests that the CRISPR-Cas9 system can avoid off-target effects in AdV genomes by careful design and selection [36]. Similarly, when a HSV1 mutant was generated using the CRISPR-Cas9 system, the gRNA-206 was aligned with HSV1 genome and no apparently significant homologous sequences outside the target region was found. Additionally, DNA sequencing of potential off-target regions found no mutations, indicating that off-target effects on the HSV1 genome can be avoided when the CRISPR-Cas9 system is used to edit its genome [36].

When CRISPR-Cas9 was used to edit VV genomes, all gRNAs were aligned with the VV genome and no potential off-target regions were found, thus, proving again, that the off-target effect can be avoided by carefully designing the gRNAs [18,37]. Due to the relatively small size of viral genomes in comparison to cellular genomes, it is much easier to avoid any potential off-targets effects when choosing gRNAs, making the CRISPR-Cas9 system an attractive approach to engineer mutant viruses [18].

## 5. Using CRISPR-Cas9 System to Create Mutant Oncolytic VV

Oncolytic viruses can be developed by attenuating and modifying viruses and/or arming the virus with a therapeutic gene [5,8,9,18,37,58,59,60,61,62,63]. The deletion of the TK gene in VV increases the selectivity such that the virus replicates preferentially in cancer cells. Thus, using an oncolytic virus or a virus as a vector for vaccines has a better safety profile [64,65,66,67,68]. The TK gene was replaced with the RFP gene using the CRISPR-Cas9 system with a greater than 90% rate in the RFP positive plaques [37], thus making the CRISPR-Cas9 system an attractive option for modification of VV. Indeed, the N1L deletion and A46R deletion viruses were created with high efficiency by the CRISPR-Cas9 system as well [18].

A computerized database for gRNA target regions on both DNA strands of viral genome of VV was generated based on the contents of A and T in the target DNA sequences [18] following the principle described previously [47]. Almost every vaccinia virus gene can be targeted by gRNA, albeit many genes can be targeted by multiple gRNAs [18]. Some gRNAs are more effective than others [18,37], therefore, it is important to test two or three gRNAs to identify the best one. The gRNA target region with off-target binding region(s) can be ruled out by aligning the gRNA target region against the VV genome [18]. The gRNA target site should be between right and left arms of the repair donor vector on the target gene (Figure 1) [18,37].

The length of the gRNA target region can be 19 bp or 20 bp for editing the genome of VV (Figure 1). The U6 promoter initiates the transcription from G on the DNA sequences [69]. The addition of a G to the gRNA, which contains a 19mers target sequence and lacks the G at the 5′ end, proved to be working [18]. An extra G can be added to the 20mers target sequence containing a G at the start [37].

The EGFP or RFP can be used as the recombinant vaccinia virus purification marker [18,37]. However, RFP is a much brighter marker than EGFP, RFP signal can be seen easily 24 h post-infection of VV, whereas a strong EGFP expression can only be seen 48 h post-infection of vaccinia virus [18]. Therefore, RFP is a preferred purification marker for purifying recombinant VV (Figure 1) [37].

A repair donor vector carrying a marker gene, such as EGFP or RFP, is required to create a mutant VV. Such a repair donor vector can carry a therapeutic gene (Figure 1 and Figure 2) [18,37]. The length of right arm and left arm can be varied from between 300 bp to the size of 500 bp or 600 bp (Figure 1) [18,37]; 500 bp to 600 bp is a better choice as it may increase the efficiency of homologous recombination (Figure 1) [18,37]. Both arms can span the target gene or slightly overlap with the target gene up to 50 bp within the target gene.

## 6. Summary

The CRISPR-Cas9 system has been used successfully in creating mutations with high efficiency in the target region of AdV and HSV-1 [36], which indicates this genome editing system can be an attractive approach to generate mutations in large viral DNA genomes. A mutant HSV virus with EGFP [40] and VV with or without RFP were created with high efficiency and accuracy by using the CRISPR-Cas9 system [18,37].

Another potential use of the CRISPR-Cas9 system is to elucidate as yet unknown functions of specific viral genes, particularly within the VV as almost every gene of VV can be targeted by gRNA guided Cas9 [18]. A mutant vaccinia virus with deletion in specific region can be easily created with the incorporation of a reporter gene (such as RFP or EGFP) in the presence of a combination of repair donor DNA, gRNA and Cas9. It is inevitable that the CRISPR-Cas9 system will play a large part in the development of future generation oncolytic viruses, as well as basic research into viral biology.
